# Chronic diseases associated with increased likelihood of hospitalization and mortality in 68,913 COVID-19 confirmed cases in Spain: A population-based cohort study

**DOI:** 10.1371/journal.pone.0259822

**Published:** 2021-11-12

**Authors:** Antonio Gimeno-Miguel, Kevin Bliek-Bueno, Beatriz Poblador-Plou, Jonás Carmona-Pírez, Antonio Poncel-Falcó, Francisca González-Rubio, Ignatios Ioakeim-Skoufa, Victoria Pico-Soler, Mercedes Aza-Pascual-Salcedo, Alexandra Prados-Torres, Luis Andrés Gimeno-Feliu, on behalf of the PRECOVID Group

**Affiliations:** 1 EpiChron Research Group, Aragon Health Sciences Institute (IACS), IIS Aragón, Miguel Servet University Hospital, Zaragoza, Spain; 2 Health Services Research on Chronic Patients Network (REDISSEC), ISCIII, Madrid, Spain; 3 Preventive Medicine and Public Health Teaching Unit, Miguel Servet University Hospital, Zaragoza, Spain; 4 Delicias-Sur Primary Care Health Centre, Aragon Health Service (SALUD), Zaragoza, Spain; 5 Aragon Health Service (SALUD), Zaragoza, Spain; 6 Drug Utilization Work Group, Spanish Society of Family and Community Medicine (semFYC), Barcelona, Spain; 7 WHO Collaborating Centre for Drug Statistics Methodology, Department of Drug Statistics, Division of Health Data and Digitalisation, Norwegian Institute of Public Health, Oslo, Norway; 8 Torrero-La Paz Primary Care Health Centre, Aragon Health Service (SALUD), Zaragoza, Spain; 9 Primary Care Pharmacy Service Zaragoza III, Aragon Health Service (SALUD), Zaragoza, Spain; 10 San Pablo Primary Care Health Centre, Aragon Health Service (SALUD), University of Zaragoza, Zaragoza, Spain; University of Catanzaro: Universita degli Studi Magna Graecia di Catanzaro, ITALY

## Abstract

**Background:**

Clinical outcomes among COVID-19 patients vary greatly with age and underlying comorbidities. We aimed to determine the demographic and clinical factors, particularly baseline chronic conditions, associated with an increased risk of severity in COVID-19 patients from a population-based perspective and using data from electronic health records (EHR).

**Methods:**

Retrospective, observational study in an open cohort analyzing all 68,913 individuals (mean age 44.4 years, 53.2% women) with SARS-CoV-2 infection between 15 June and 19 December 2020 using exhaustive electronic health registries. Patients were followed for 30 days from inclusion or until the date of death within that period. We performed multivariate logistic regression to analyze the association between each chronic disease and severe infection, based on hospitalization and all-cause mortality.

**Results:**

5885 (8.5%) individuals showed severe infection and old age was the most influencing factor. Congestive heart failure (odds ratio -OR- men: 1.28, OR women: 1.39), diabetes (1.37, 1.24), chronic renal failure (1.31, 1.22) and obesity (1.21, 1.26) increased the likelihood of severe infection in both sexes. Chronic skin ulcers (1.32), acute cerebrovascular disease (1.34), chronic obstructive pulmonary disease (1.21), urinary incontinence (1.17) and neoplasms (1.26) in men, and infertility (1.87), obstructive sleep apnea (1.43), hepatic steatosis (1.43), rheumatoid arthritis (1.39) and menstrual disorders (1.18) in women were also associated with more severe outcomes.

**Conclusions:**

Age and specific cardiovascular and metabolic diseases increased the risk of severe SARS-CoV-2 infections in men and women, whereas the effects of certain comorbidities are sex specific. Future studies in different settings are encouraged to analyze which profiles of chronic patients are at higher risk of poor prognosis and should therefore be the targets of prevention and shielding strategies.

## Introduction

The coronavirus disease 2019 (COVID-19) pandemic was declared a Public Health Emergency of International Concern by the World Health Organization in March 2020 and, since then, SARS-CoV-2 has affected more than 150 million people and has caused over 3.2 million deaths worldwide [1].

Clinical characteristics among COVID-19 patients vary greatly, especially with age [2]. Most cases, in particular in children and younger adults, manifest as asymptomatic or mild infections, while others, typically older adults or patients with comorbidities, can develop severe to critical presentations with acute respiratory distress syndrome (ARDS) and even death [2, 3]. Apart from age, the concurrence of specific chronic conditions has been amply linked through metanalytical data to increased risk of severe infection, with chronic lung and kidney diseases, obesity, diabetes, hypertension, or malignancy as some of the main examples [4, 5]. COVID-19 has also been associated with intense multisystemic inflammation and an uncontrolled immune response, leading to cardiovascular complications and poorer outcomes, more so in patients with underlying comorbidities [6, 7].

The definition of severity for COVID-19 infection differs across the existing literature, as evidenced by the systematic reviews conducted by Flook et al. and Li et al., where some authors followed the international guidelines for community acquired pneumonia or clinical criteria to classify severe cases (acute respiratory distress syndrome, tachypnea, hypoxemia, pulmonary imaging, multi-organ failure), while others considered outcomes such as intensive care unit (ICU) admission, invasive ventilation support or death, among others [5, 8]. Clinical factors, along with other individual features such as functional capacity and autonomy, are key in determining COVID-19 severity in routine clinical practice [9]. However, indicators such as mortality and hospitalization can serve as proxy, and aid in decision-making for the allocation of expected resources and the development of mitigation and prevention strategies at a health system level, which also have an influence on health results in infected patients [10].

In our previous study on comorbidity in COVID-19 patients within the PRECOVID cohort we used all-cause mortality as the only outcome to measure severity [11]. The epidemiological situation at the time, with an overburdened healthcare system that could not assume all the necessary hospitalizations, and a lack of overall testing capacity, impeded the use of other relevant indicators like hospital and ICU admissions during follow-up. With the improvement of testing capacity, allowing for microbiological confirmation of all COVID-19 cases, and less pressure in hospitals thanks to large-scale preventive measures (i.e., use of facemasks, confinements, quarantines, limited social reunions and contact tracing), current data on admission can be reliably studied along with mortality as an indicator of severity. While many studies focus on in-hospital mortality alone, often with self-reported diagnoses of specific chronic conditions [12], PRECOVID is a population-based cohort that offers a holistic view of baseline comorbidity profiles and health outcomes, diagnosed and recorded in electronic health records by healthcare professionals in both in- and out-patient settings.

The objective of the present study is to determine the demographic and clinical factors, particularly baseline chronic conditions, associated with an increased risk of severity in COVID-19 patients, operationalized through a combined outcome of hospitalization and all-cause mortality.

## Methods

### Design and study population

We conducted a retrospective, observational study in the PRECOVID cohort [10], an open cohort of all the individuals with confirmed SARS-CoV-2 infection in the Spanish region of Aragon (reference population, 1.3 million inhabitants). Cases were confirmed using PCR and antigen-detection tests following the national protocol from the Ministry of Health on early detection, surveillance and control of COVID-19. We analyzed all 68,913 cases that were confirmed in Aragon during enrollment, which spanned from 15 June 2020 to 19 December 2020 for this study. We excluded patients diagnosed during the first three months of the pandemic (March through May 2020) as testing capacity in our region was limited at the time.

Each patient was followed for up to 30 days from the date of inclusion in the cohort (index date) or until the date of death within that period in order to analyze the effect of patients’ baseline characteristics on infection severity. The index date was defined by the date of the confirmatory test result. Infections were categorized as mild or moderate-to-severe, referred to from now on as severity, based on the need for hospital admission (including admissions to the Intensive Care Unit—ICU) and 30-day mortality.

The Clinical Research Ethics Committee of Aragón approved the research protocol for this study (CEICA; PI20/226) and waived the requirement to obtain informed consent from participants due to the epidemiological nature of the project and the use of anonymized data, presented only at an aggregated level.

### Variables of study and data sources

The PRECOVID study cohort analyzes COVID-19 clinical and epidemiological characteristics through the linkage of real-world data from electronic health records (EHRs) and clinical-administrative databases. Patient data was obtained from the Aragon Health System by associating information from the users’ health database, EHRs, and an ad hoc COVID-19 registry, at a person level and in a pseudo-anonymized form.

We studied the following baseline demographic and clinical characteristics for each participant at the time of inclusion in the cohort: sex, age, type of residence area (rural vs. urban), and all baseline chronic diseases registered in EHRs. Primary care diagnoses were coded using the International Classification of Primary Care, First Edition (ICPC–1), and mapped to ICD-9-CM using a system developed to codify temporary disability [13]. The codes were subsequently sorted into 226 clinically relevant categories using the Clinical Classifications Software (CCS) [14], and 153 of them were classified as chronic via the Chronic Condition Indicator Software [15]. The group’s clinical experts recoded the final list into 156 diagnostic categories with minor adjustments to facilitate their clinical interpretation. Conditions present at least during the last 12 months and meeting one or both of the following criteria were included in the analysis: a) entails limitations on self-care, independent living, and social interactions; b) requires of ongoing interventions using medical products, services, and special equipment.

During follow-up, we analyzed whether the patient experienced severe infection, defined as hospitalization (including admission to the ICU) or all-cause mortality, or mild infection (rest of the cases). Patients were followed for a maximum of 30 days from their inclusion in the cohort to give enough time for the studied outcomes to occur. Events taking place after the set follow-up period were considered difficult to attribute to COVID-19 infection seeing as the exact cause of death was not available in our registries. Regarding hospitalizations, only admissions occurring within 15 days of the index date were considered a consequence of the infection. Since some patients were diagnosed only after admission, we also accounted for hospitalizations occurring up to 15 days before the index date.

### Statistical analysis

We described the sociodemographic and clinical characteristics of all patients from the study as means accompanied by their respective standard deviations or as absolute and relative frequencies and proportions, stratified by sex and by severity of infection.

We performed logistic regression models to analyze the association between the presence of each baseline chronic disease and the occurrence of severe infection. We used logistic regression instead of Cox regression since we had the date of inclusion in the cohort (date of the first confirmatory test result), but not the exact date of infection; moreover, follow-up was truncated at 30 days after the index date for all individuals. We calculated age-adjusted odds ratios (ORs) accompanied by their respective 95% confidence intervals (CI). Analysis was stratified by sex. Only chronic diseases with a prevalence equal to or higher than 0.5% in each sex and with a significant association with infection severity were presented in tables; the rest can be found as Supporting information.

Finally, a multivariate logistic regression analysis was performed to identify which chronic comorbidities were significantly associated with higher infection severity when studied in combination, with age as an added explanatory variable. The multivariate analysis was performed using a stepwise method via the STEP function from the stats package, and was based on the Akaike Information Criterion (AIC) with an exit p-value of 0.05. AIC is preferable as a criterion for model selection in situations where the model is intended for predictive purposes. All statistical analyses were conducted in R 3.6.3 and Stata 14. Statistical significance was set at p < 0.05.

## Results

A total of 68,913 individuals were included in the study, with a mean age of 44.4 years. 53.2% were women, 39.1% lived in rural areas and 58.6% presented multimorbidity, with an average of 2.89 chronic diseases at infection (Table 1). During follow-up, 5885 (8.5%) individuals showed severe infection as defined in our study (88.6% of them were hospitalized, 5.52% were admitted to the ICU, and 27.2% died). The risk of severe infection was 71.1% higher in men compared to women and consistently increased with age in both sexes, with patients aged 80 years and over showing 8.23 (7.64–8.87) times higher risk than those in the 45–64 years age group.

**Table 1:** Demographic and clinical characteristics of individuals with confirmed COVID-19 infection and effect on disease severity (defined as mild or moderate-to-severe according to hospital admission and mortality).

Characteristics	All (n = 68,913)			P value	Women (n = 36,682)			P value	Men (n = 32,231)			P value
	Mild^a^ (x = 63,028)	Moderate-to-severe^b^	OR^c^ (95% CI^d^)		Mild	Moderate-to-severe (n = 2778)	OR (95% CI)		Mild	Moderate-to-severe	OR (95% CI)	
		(n = 5885)			(n = 33,904)				(n = 29,124)	(n = 3107)		
**Sex, men** (n, %)	29,124 (46.2)	3107 (52.8)	1.71 (1.61–1.82)	<0.001	-	-	-	-	-	-	-	-
**Age** (mean, sd^e^)	41.9 (22.7)	71.6 (18.7)	1.07 (1.06–1.07)	<0.001	43.3 (23.3)	73.9 (19.2)	1.06 (1.06–1.06)	<0.001	40.3 (21.9)	69.6 (18.0)	1.07 (1.07–1.07)	<0.001
**Age intervals** (n, %)												
≤14	8075 (12.8)	47 (0.80)	0.07 (0.06–0.10)	<0.001	4020 (11.9)	16 (0.58)	0.07 (0.04–0.12)	<0.001	4055 (13.9)	31 (1.00)	0.07 (0.05–0.10)	<0.001
15–44	26,367 (41.8)	469 (7.97)	0.23 (0.21–0.25)	<0.001	13,902 (41.0)	234 (8.42)	0.31 (0.27–0.37)	<0.001	12,465 (42.8)	235 (7.56)	0.18 (0.16–0.21)	<0.001
45–64	18,358 (29.1)	1422 (24.2)	Reference	-	9844 (29.0)	530 (19.1)	Reference	-	8514 (29.2)	892 (28.7)	Reference	-
65–79	5905 (9.37)	1427 (24.2)	3.11 (2.88–3.37)	<0.001	3118 (9.20)	579 (20.8)	3.45 (3.05–3.91)	<0.001	2787 (9.57)	848 (27.3)	2.90 (2.62–3.22)	<0.001
≥80	4323 (6.86)	2520 (42.8)	8.23 (7.64–8.87)	<0.001	3020 (8.91)	1419 (51.1)	8.73 (7.84–9.72)	<0.001	1303 (4.47)	1101 (35.4)	8.07 (7.26–8.97)	<0.001
**Rural area**^f^ (n, %)	24,561 (39.0)	2415 (41.0)	0.87 (0.82–0.92)	<0.001	12,822 (37.8)	1097 (39.5)	0.82 (0.75–0.89)	<0.001	11,739 (40.3)	1318 (42.4)	0.91 (0.84–0.99)	0.033
**Chronic diseases**^g^ (mean, sd)	2.7 (2.8)	5.2 (3.8)	1.04 (1.03–1.05)	<0.001	3.1 (3.0)	5.8 (3.9)	1.03 (1.02–1.04)	<0.001	2.2 (2.4)	4.6 (3.5)	1.05 (1.04–1.07)	<0.001
**Follow-up outcome**												
**Hospital adm**.^h^ (n, %)	-	5214 (88.6%)	-	-	-	2441 (87.9%)	-	-	-	2773 (89.3%)	-	-
**ICU**^i^ **adm**. (n, %)	-	325 (5.52%)	-	-	-	107 (3.85%)	-	-	-	218 (7.02%)	-	-
**Death**^j^ (n, %)	-	1598 (27.2%)	-	-	-	791 (28.5%)	-	-	-	807 (26.0%)	-	-

^a^Defined as individuals without hospital admission or mortality during the follow-up period;

^b^Defined as individuals admitted to hospital or dead during the follow-up period;

^c^Odds ratio of moderate-to-severe infection adjusted by categorical age;

^d^95% confidence interval;

^e^standard deviation;

^f^Versus urban area, according to patients’ administrative health area;

^g^From a list of 153 chronic conditions;

^h^Admission to hospital during 15 days before/after the index date;

^i^Admission to the Intensive Care Unit;

^j^30-day all-cause mortality.

**Fig 1:**
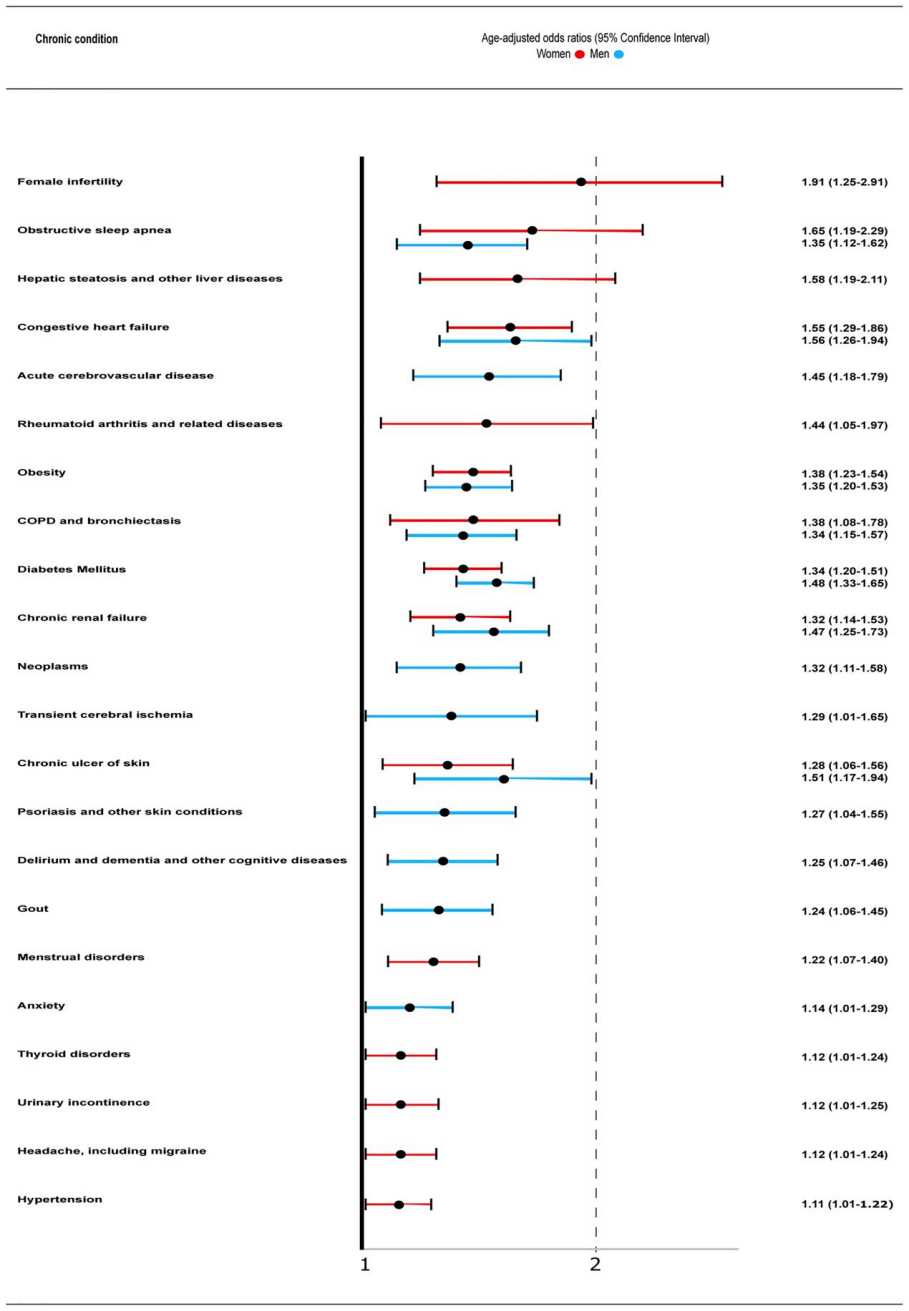
Baseline chronic comorbidities associated with moderate-to-severe infection, defined as the combined outcome of hospital admission and 30-day all-cause mortality, in women (red) and men (blue) with COVID-19 infection. COPD, chronic obstructive pulmonary disease.

Baseline chronic diseases associated the most with severe infection in COVID-19 infected women when studied separately and after controlling by age were (OR [95% CI]) female infertility (1.91 [1.25–2.91]), obstructive sleep apnea (OSA; 1.65 [1.19–2.29]), hepatic steatosis and other liver diseases (1.58 [1.19–2.11]), congestive heart failure (1.55 [1.29–1.86]), rheumatoid arthritis (1.44 [1.05–1.97), obesity (1.38 [1.23–1.54]), chronic obstructive pulmonary disease (COPD; 1.38 [1.08–1.78]), diabetes mellitus (1.34 [1.20–1.51]), and chronic renal failure (1.32 [1.14–1.53]), among others (Fig 1). The complete list of basal chronic conditions in infected women along with their prevalence and association with infection severity is presented as S1 Table.

In men, baseline chronic conditions associated the most with higher infection severity when studied separately were (OR [95% CI]) congestive heart failure (1.56 [1.26–1.94]), chronic ulcers of skin, (1.51 [1.17–1.94]), diabetes mellitus (1.48 [1.33–1.65]), chronic renal failure (1.47 [1.25–1.73]), acute cerebrovascular disease (1.45 [1.18–1.79]), obesity (1.35 [1.20–1.53]), obstructive sleep apnea (1.35 [1.12–1.62]), COPD (1.34 [1.15–1.57]), urinary incontinence (1.33 [1.15–1.54]), and neoplasms (1.32 [1.11–1.58]), among others (Fig 1). The complete list of chronic diseases and their association with infection severity in men is presented as S2 Table.

The models assessing the likelihood of severe COVID-19 infection based on sex, age and comorbidity at baseline showed that age was the most influencing factor, with an increased average risk of 6.19 in men and 8.19 in women aged 80 and over compared to their 45-64-year-old counterparts (Table 2). Nine conditions in men and nine in women were significant in the models (OR men, OR women), with congestive heart failure (1.28, 1.39), diabetes mellitus (1.37, 1.24), chronic renal failure (1.31, 1.22) and obesity (1.21, 1.26) increasing the likelihood of severe infection in both sexes. Additionally, chronic ulcers of the skin (1.32), acute cerebrovascular disease (1.34), COPD (1.21), urinary incontinence (1.17) and neoplasms (1.26) in men, and female infertility (1.87), OSA (1.43), hepatic steatosis (1.43), rheumatoid arthritis (1.39) and menstrual disorders (1.18) in women were also associated with higher risk of severity.

**Table 2:** Models of likelihood of moderate-to-severe COVID-19 infection according to baseline demographic and clinical variables of the individuals with laboratory-confirmed infection, by sex.

Sex	Women (n = 36,682)				Men (n = 32,231)			
Variables	OR^b^	95% CI^a^ Lower limit	95% CI Upper limit	p value	OR	95% CI Lower limit	95% CI Upper limit	p value
(Intercept)	0.05	0.04	0.05	<0.001	0.09	0.09	0.10	<0.001
Age, 0–14 years	0.08	0.05	0.14	<0.001	0.08	0.06	0.12	<0.001
Age, 15–44 years	0.32	0.28	0.38	<0.001	0.20	0.17	0.23	<0.001
Age, 65–79 years	3.33	2.92	3.81	<0.001	2.45	2.20	2.73	<0.001
Age, ≥80 years	8.19	7.24	9.26	<0.001	6.19	5.47	6.99	<0.001
Congestive heart failure	1.39	1.16	1.68	<0.001	1.28	1.03	1.60	0.028
Diabetes Mellitus	1.24	1.10	1.40	<0.001	1.37	1.23	1.53	<0.001
Chronic renal failure	1.22	1.05	1.42	0.011	1.31	1.11	1.55	0.002
Obesity	1.26	1.13	1.42	<0.001	1.21	1.06	1.37	0.003
Female infertility	1.87	1.22	2.85	0.004	-	-	-	-
Obstructive sleep apnea	1.43	1.03	2.00	0.035	-	-	-	-
Hepatic steatosis and other liver diseases	1.43	1.07	1.91	0.016	-	-	-	-
Rheumatoid arthritis and related disease	1.39	1.01	1.92	0.042	-	-	-	-
Menstrual disorders	1.18	1.03	1.35	0.016	-	-	-	-
Chronic ulcer of skin	-	-	-	-	1.32	1.02	1.71	0.032
Acute cerebrovascular disease	-	-	-	-	1.34	1.09	1.65	0.006
COPD^c^ and bronchiectasis	-	-	-	-	1.21	1.03	1.42	0.022
Urinary incontinence	-	-	-	-	1.17	1.00	1.36	0.044
Neoplasms	-	-	-	-	1.26	1.06	1.51	0.010

^a^95% confidence interval;

^b^Odds ratio;

^c^Chronic obstructive pulmonary disease.

## Discussion

This large-scale population-based study describes the clinical and demographic characteristics associated with an increased risk of severity in 68,913 individuals with confirmed SARS-CoV-2 infection. Our findings show that, apart from old age and male sex, specific baseline cardiovascular and metabolic conditions are common risk factors for severe infection in men and women, while other comorbidities are sex-specific.

Our analyses reasserted that age is by far the most influencing factor on the vulnerability of older people to develop more severe forms of COVID-19, most notoriously when crossing the threshold of the 80s, highlighting the need for targeted prevention and early detection strategies in older age groups [16, 17]. Men were at higher risk of severity than women, a fact that has been amply reported and attributed to various causes such as lifestyle differences (e.g. smoking) or hormone differences in inflammatory processes [18].

Cardiovascular and metabolic comorbidities (i.e. obesity, diabetes, congestive heart failure and chronic renal failure) increased the risk of severe infection in both sexes even when adjusting by age and the remaining significant comorbidities, as described in previous studies [19–24]. Patients with these conditions seem to be predisposed to potentially serious COVID-19 infections through common pathophysiological pathways, with chronic systemic inflammation at their center [7]. Key aspects include ACE-2-mediated endothelial injury, blood flow stasis due to immobilization, and a state of hypercoagulability induced by prothrombotic circulating factors, which contribute to the occurrence of coagulopathies and thromboembolic events, markedly increasing the risk of severe outcomes [25–29]. Arterial hypertension was abundantly associated with poor health outcomes after COVID-19 infection in early studies, but was usually reported along with potentially confounding effects such as age and other chronic conditions [30]. In our study, hypertension was not a risk factor for severity in men, and lost its significance when adjusting by the rest of comorbidities in women, supporting the theory that it entails higher risk of severity only in association with other diseases but not as an isolated condition [31].

Vulnerable ACE2-expressing tissues include the endothelium, heart, kidneys, lungs, liver, brain, thyroid, testis, and small intestine. ACE2 expression levels and immune signatures depends on the organ or tissue, and is also sex- and age-dependent, partly explaining distinct symptoms and severity [32]. Chronic respiratory diseases like COPD and OSA have been previously identified as risk factors for severe outcomes in COVID-19 patients, and have also been associated with higher risk of thrombosis, coagulopathy and cardiovascular morbidity [22, 33–40]. In our study we found similar results but, when adjusting by the rest of comorbidities, COPD only remained significant in men, and OSA only in women.

In addition to COPD and OSA, some chronic conditions were independent predictors of severity only in one sex. In men, neoplasms and acute cerebrovascular disease were independently associated with COVID-19 severity. Both entities had been previously identified as risk factors for poor prognosis [20–23]. Chronic ulcers of the skin and urinary incontinence, usually linked to serious states of functional impairment, were also significantly associated with higher risk in our age-adjusted analysis, concurring with our previous study [11]. Anxiety, as well as dementia, delirium and other cognitive disorders were additional risk factors for severity, also only in men in our case, and further research could shed light on the benefits of including neuropsychological evaluation and rehabilitation during and after COVID-19 infection, as has been stated in the literature [41, 42]. In women, hepatic steatosis and rheumatoid arthritis were risk factors for severe outcomes. Although the association of liver disease with a more severe progression of COVID-19 has been described [43], the role of rheumatoid arthritis remains unclear in women, highlighting the importance of age, comorbidity, disease activity and pharmacological treatments in future studies [44, 45].

An unexpected finding was the increased risk of severe infection in women with clinical history of infertility or menstrual disorders. It is well known that assisted reproduction techniques, especially ovarian stimulation, are associated with a higher risk of arterial and venous thromboembolic complications [27]. Further studies are encouraged in the future to specifically address the potential mid- and/or long-term effects of these conditions and its treatments on COVID-19 infection severity. The interpretation of the association of menstrual disorders with severity is challenging, especially considering how varied the pathophysiology of the processes included under this denomination is (i.e., polycystic ovary, dysmenorrhea, metrorrhagia), and would also require more targeted and exhaustive studies.

Regarding thyroid disorders, we found higher risk of severe outcomes in women, though the effect was lost when adjusting by the rest of comorbidities. To date, there is no evidence suggesting that these disorders increase the risk of poor prognosis in COVID-19 patients [46]. Notwithstanding, thyroid disfunction does influence the regulation of immunity, particularly if it is cell-based. Low concentrations of thyroid hormones have been associated with increased mortality in patients with serious infectious diseases [46, 47] and could have been partly responsible for some of the more severe COVID-19 infections, seeing as 80% of those diagnosed with thyroid disorders had hypothyroidism. As far as we know, only two previous studies have analysed this hypothesis, and although both of them ruled out any significant association between hypothyroidism and COVID-19 infection severity, further studies are warranted [46, 48].

Due to their low prevalence (between 0.1–0.5%), some chronic conditions were not presented in the Results section of the manuscript despite statistical significance (available as supplementary tables), and their potential impact on infection severity may have not been sufficiently analyzed. In the case of men, two inflammatory conditions were associated with higher severity: rheumatoid arthritis (in line with what was observed in women) and polymyalgia. It has been reported that autoimmune diseases are not significantly associated with severe COVID-19 progression [49], and poor outcomes could simply be linked to older age and comorbidity [45], though one study did find a 39% increase in the adjusted risk of hospitalization and mortality in a sample of mostly male patients with COVID-19 infection and rheumatoid arthritis [50]. In women, we found a high risk of severe COVID-19 outcomes in patients with HIV infection and, although similar findings have been published in the literature, further studies are needed to better assess this risk taking into account disease control, treatment, analytical blood parameters, and comorbidity [51–53].

### Strengths and limitations

The main strength of our study is its population-based nature. It includes all the confirmed COVID-19 cases in the region, with a sample size of nearly 70,000 individuals. Another key feature of our study is that baseline comorbidities were not limited to the most prevalent or to those self-reported by patients, but were based on a comprehensive analysis of all the chronic diagnoses registered by physicians in their EHRs instead. Furthermore, we used open-access software with an open algorithm to manage chronic conditions in the analyses, facilitating reproducibility and comparability. Our study, however, is not free of limitations. Some sociodemographic variables, such as socioeconomic status and ethnicity, or clinical variables like laboratory tests results, acute respiratory distress, multi-organ failure, pharmacological treatments, or genetic profiles could have influenced infection severity or aided in the interpretation of the results, but were not available in our cohort. Moreover, the causes of hospitalization and/or death were also unavailable, and we were therefore unable to directly attribute the observed outcomes to the infection. To minimize these limitation, we assessed both outcomes in a limited time window based on clinical criteria, and excluded COVID-19 patients from the first three months of the pandemic where access to diagnostic tests and in some cases medical care was more limited. Finally, though we conducted comorbidity-adjusted multivariate logistic regression analyses, each chronic condition contributed independently to estimate the associated risk of severity. We know, however, that patients with more severe forms of infection are usually affected by more than one chronic disease, as was the case in our study (81% were multimorbid, with an average of 5.2 chronic conditions). Considering that chronic diseases tend to cluster into multimorbidity patterns, future studies should focus on the combined burden of coexisting conditions and the effects of their interactions on COVID-19 outcomes, as well as on the consequences of COVID-19 on patients and the healthcare system.

## Conclusions

This population-based study reasserts that old age is the most influencing factor on COVID-19 severity and contributes to the ongoing discussion on the effects of comorbidity on the evolution of the infection. The presence of specific cardiovascular and metabolic conditions (i.e. heart failure, diabetes mellitus, obesity and chronic renal failure) consistently increased the risk of severe SARS-CoV-2 infections in men and women. Results also reveal sex differences in certain disorders, such as neoplasms and acute cerebrovascular disease in men or thyroid disorders and hepatic steatosis in women, whose effects on COVID-19 severity have been less studied to date. More in-depth research on the impact of each disease and their combinations is encouraged to ascertain whether COVID-19 could act as a trigger for negative health outcomes in patients with specific multimorbidity profiles.

## Supporting information

S1 Table Complete list of baseline chronic comorbidities of women with COVID-19 infection and association with moderate-to-severe infection, defined as the combined outcome of hospital admission and 30-day all-cause mortality. (XLSX)

S2 Table Complete list of baseline chronic comorbidities of men with COVID-19 infection and association with moderate-to-severe infection, defined as the combined outcome of hospital admission and 30-day all-cause mortality. (XLSX)
